# Hand-Carried Ultrasonography Instrumentation in the Diagnosis of Temporomandibular Joint Dysfunction

**DOI:** 10.3390/mps4040081

**Published:** 2021-11-06

**Authors:** Marco Severino, Silvia Caruso, Sofia Rastelli, Roberto Gatto, Tommaso Cutilli, Laura Pittari, Alessandro Nota, Simona Tecco

**Affiliations:** 1 Department of Life, Health and Environmental Science, University of L’Aquila, 67100 L’Aquila, Italy; marcoseverino1@gmail.com (M.S.); silvia.caruso@cc.univaq.it (S.C.); sofiarastelli3@gmail.com (S.R.); roberto.gatto@cc.univaq.it (R.G.); tommaso.cutilli@cc.univaq.it (T.C.); 2 I.R.C.C.S. San Raffaele Hospital, Vita-Salute San Raffaele University, 20132 Milan, Italy; laura_pittari@hotmail.it (L.P.); nota.alessandro@hsr.it (A.N.)

**Keywords:** hand-carried ultrasonography instrumentation, temporomandibular joint, internal derangement

## Abstract

Internal derangement (ID) in the temporomandibular joint (TMJ) is defined as a mechanical problem of the joint that interferes with its function. It is attributed to an abnormal interaction among the articular disc, condyle, and joint eminence. The aim of this study is to evaluate diagnostic efficacy of non-invasive hand-carried ultrasonography instrumentation (US) to provide high-level images for a correct diagnosis of ID. Twenty-eight ID patients, 15 female and 13 males, were examined both clinically and by MRI images in order to achieve a diagnosis of ID (using Helkimo index). Then, they were submitted to US examination with a 12 MHz transducer by using hand-carried instrumentation by a clinician that was blind to their diagnosis and clinical data. TMJ US examination was performed with the mouth closed and mouth open, with proper technique. Each position was then evaluated with two different orientations of the transducer. US showed acceptable results in identifying bone structures. Lower values of diagnostic efficacy were obtained for disc position during joint movements with respect to MRI images. MRI still represents the gold standard for the identification of joint structures. If not corroborated by clinical and anamnestic data, the diagnostic efficacy of US in identifying the position of the disc during opening and closing jaw movements appears limited than compared to MRI.

## 1. Introduction

Temporomandibular joint (TMJ) is a synovial articulation between the mandibular condyle and the glenoid fossa in the temporal bone. TMJ disorders (TMD) constitute structural and/or functional disorders that affect TMJ, masticatory muscles, and related structures. These disorders may present with clinical signs such as articular noises, TMJ pain, and/or limitations in opening and closing the mouth [1].

Among TMDs, internal derangement (ID) is defined as a mechanical problem of the joint, characterized by disc dislocation, that interferes with the function of the joint itself [1,2]. This is attributed to an abnormal interaction of the articular disc, condyle, and joint eminence. 

Anterior disc displacement (ADD) is the most common ID condition and is usually divided into two categories: with reduction and without reduction. Each category has special features. In ADD with reduction, the disc locates anterior to its normal position when the mouth closes. However, in the open mouth position, it returns to its normal position while it remains anteriorly displaced in ADD without reductions. Therefore, the examination should include both closed and open mouth positions [2,3,4].

ID diagnosis is based on anamnestic interview about pain and dysfunction and clinical examination, aimed to assess mandibular movement [2,3]. For a certain diagnosis, it is crucial that there is a visualization of articular disc both in the closed and opened mouth positions [5,6,7,8,9,10].

Today the gold standard for disc visualization is based on MRI images, taken with closed and opened mouth, which are mostly static images taken with an uncomfortable and high-cost instrumentation. In order to overcome these limits, a promising tool seems to be represented by ultrasonography instrumentation (US) [11,12,13,14,15,16,17,18]. This non-ionizing imaging method is less expensive, more comfortable to the patient, even dynamic, and could be easily used in a dental setting [13,14,15,16,17].

US is based on high frequency emitted pulses and subsequent echoes detected by a transducer placed in contact with the patient skin, acquiring static and dynamic images in real time. In general, the pulses frequency ranges from 2 MHz to 15 MHz depending on the depth of the anatomic region to be evaluated. For TMJ, the protocol includes longitudinal and transverse scans using transducers with frequencies ranging from 7.5 MHz to 15 MHz [18,19,20]. Static or dynamic evaluations can be performed while the mouth is closed or opened. 

TMJ region consists of several structures that reflect sound waves differently [10]. Bone tissue, represented by the head of the condyle and the joint eminence, is generally hypoechoic (poorly reflected by sound waves) and appears black in ultrasound images [13,14,15,16,17]. However, bone margin is hyperechoic (highly reflected by sound waves) and appears white in ultrasound images [13,14,15,16,17]. Connective tissues, represented by joint capsule, retrodiscal zone, and muscles that are lateral pterygoid and masseter muscles, are isoechoic (intermediate reflex of sound waves) and appear heterogeneous and gray in ultrasound images [13,14,15,16,17].

The surface of joint capsule, as well as the surface of muscles, highly reflect the sound waves, thus generating a hyperechoic (white) line. [13,14,15,16,17] Empty space and water, such as the upper and lower joint spaces, are hypoechoic and appear black on ultrasound images [13,14,15,16,17]. However, these anatomical cavities are virtual, as opposing surfaces that are in contact, and are usually undetectable unless there is an effusion [13,14,15,16,17].

Visualization of deep structures, such as joint disc, is difficult due to the absorption of waves by the lateral portion of condylar head and zygomatic process of temporal bone [13,14,15,16,17]. On ultrasound, the disc appears as a thin homogeneous and hypo-isoechoic band [13,14,15,16,17].

**Figure 1:**
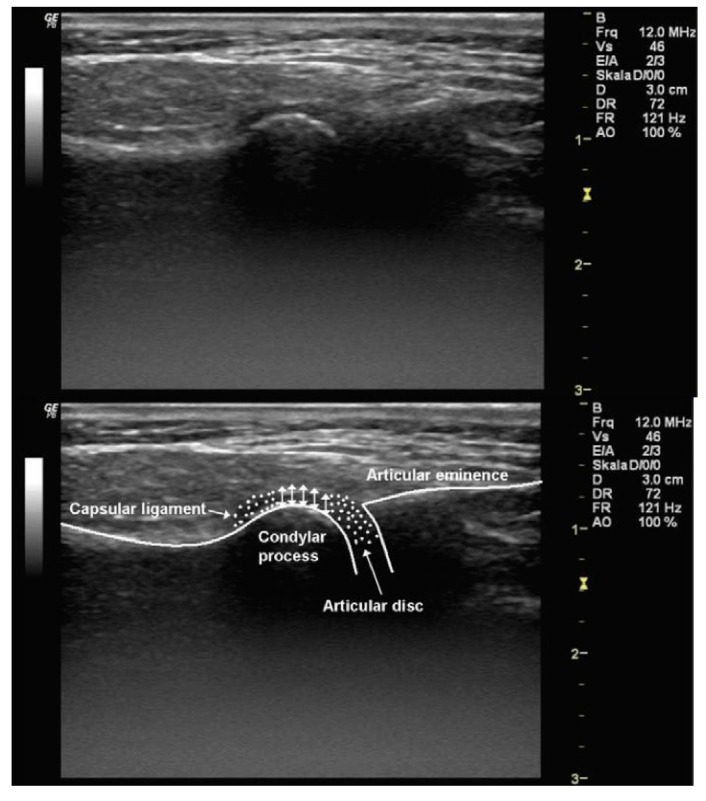
The image evidence hypoechoic bone tissue, hyperechoic bone surface, and hypo-isoechoic connective tissues.

Figure 1 reports an ultrasound image of a TMJ. 

In evaluating closed mouth images, disc position is considered normal if its intermediate zone is between the anterior-superior part of condyle and posterior-inferior part of joint eminence [10]. The discs located forward to this position are considered displaced into an anterior direction [10].

In evaluating open mouth images, disc position is considered normal if its intermediate zone is between condyle and joint eminence [10]. The discs displaced in the forward direction represent evidence of internal derangement [10].

Joint effusions can be detected indirectly by measuring the distance between the two joint surfaces [10].

Melis et al. assessed that ultrasonography sensitivity ranged from 13–100% for the evaluation of disc displacement (DD), from 70.6–83.9% for the evaluation of joint effusion (JE), and from 70–94% for the evaluation of condylar erosion (CE). Specificity ranged from 62–100% for the evaluation of DD, from 73.7–100% for the evaluation of JE, and from 20–100% for the evaluation of CE. Accuracy ranged from 51.5–100% for the evaluation of DD, from 72.2–95% for the evaluation of JE, and from 67–94% for the evaluation of CE [21].

Ultrasonographic temporomandibular joint examinations should not be limited to disc displacement but should also assess degenerative changes regarding joint surfaces, joint effusion, and synovitis [22].

Today, US is often used in orthognathic surgery for arthrocentesis in order to detect disc and bone structures.

There are studies reporting acceptable diagnostic efficacy of US to detect disc displacement [18,19]. These studies are mostly based on the use of console instrumentations.

Due to high Se, Spe, Ac, PPV, and NPV found for the assessment of anterior disc position and effusion along with highly accurate measurements, ultrasound can be suggested as an adjunct to common imaging modalities in the assessment of TMJ for oral and maxillofacial radiologists due to its advantages such as the following: non-ionizing radiation, availability, ease of usage, and providing real-time rapid images at a low cost [23].

Therefore, the purpose of this study is to evaluate diagnostic efficacy of a hand-carried US (Mindray-M7 hand-carried ultrasound system, with 12 MHz linear transducer, China) relative to detecting ID alterations, specifically ADD with or without reduction. 

## 2. Materials and Methods

The present study was conducted at the Unit of maxillofacial surgery of the University of L’Aquila from January 2018 to January 2019. 

### 2.1. The Sample

The sample included 28 patients (15 female and 13 male) aged from 19 to 27 years who were recruited based on the presence of clicks and/or joint roar and of having performed an MRI of their TMJ. Demographic and diagnostic data of the subjects are summarized in Table S1. 

All the included subjects were diagnosed as affected by ID (2a E 2b, according to DC/TMD [24]) using MRI images and clinical examination. The level of TMJ pathology was classified on the base of Helkimo criteria (Helkimo anamnestic index and clinical dysfunction index) [6,7]. Each participant was informed about the aim of the study and gave her/his written consent for data publication. 

The study was conducted according to the guidelines of the Declaration of Helsinki and approved by the Institutional Review Board (or Ethics Committee) of University of L’Aquila (protocol code DR206/2013, dated 10 January 2014) which included observational studies. Informed consent was obtained from all subjects involved in the study.

### 2.2. US Examination

US examinations were performed for all patients by the same operator (the author T.C) with the Mindray-M7 hand-carried US instrumentation, using a 12 MHz linear transducer. The operator was blind to the results of clinical diagnosis and MRI images of the patients. All US examinations were performed by darkening the room to magnify the image on the PC monitor.

In order to avoid errors, the acquisitions were carried out bilaterally with a standardized sequence. All US examinations started on the left side, positioning the transducer parallel to the Frankfurt plane. The first scan was performed in the mandibular rest position with the mouth closed, and the second scan was performed in the position in maximum opening of the mouth. The position of the transducer was then modified at an angle of 60°−70° with respect to the Frankfurt plane. Thus, two scans were taken again; the first scan was performed in the rest position, with the mouth closed, and the second scan was performed in the position of maximum opening of the mouth.

### 2.3. Analysis of Data

An operator, blinded to the name of patients, reviewed the MRI images. A total of 28 × 2 TMJs were analyzed and classified on the base of condylar position, disc position, identification of joint structure, and joint dynamic movements. Then, the correspondences between MRI data and US data were compared. In particular, the number of differences were noted.

For the efficacy in recognition of the anatomical structures of the TMJ, the relative sensitivity and specificity values obtained by MRI and US were compared. In particular, sensitivity and specificity values relating to the position of the condyle, the disc, and joint dynamics were analyzed by considering MRI images as gold standard. Specificity was assessed as the percentage (on the entire sample) of false positive cases (data categorized as uncorrected with US but really correct on the base of MRI). US was considered with high specificity when false positive were <5%.

## 3. Results

Data of sensitivity and specificity are reported in Table 1.

US showed lower values for both sensitivity and specificity respect to MRI images. Low values were recorded for sensitivity in the assessment of disc position (65% of US respect to 89% of MRI).

In addition, low values of sensitivity and specificity were assessed for joint dynamics (respectively, 54% and 56% for US with respect to 76% and 76% for MRI images). 

## 4. Discussion

This study used a 12 MHz transducer and evidenced a fairly sensitive diagnostic aid in individuating TMJ structure and dynamics. It showed low values for sensitivity in the assessment of disc position (65% of US with respect to 89% of MRI) and low values of sensitivity and specificity in the assessment of joint dynamics (respectively, 54% and 56% for US with respect to 76% and 76% for MRI images). 

Previous literature evidence that there are variations across all articles for assessing disc position depending on transducer resolution [15,17,18]. Sensitivity was found to be directly proportional to the resolution of the transducer, as the increase in resolution has increased the sensitivity of the US [15,17,18]. For example, Emshoff et al. [15] used a 7.5 MHz transducer, with which the sensitivity was 41–50% and the specificity was 70%, while in other studies the transducer used was 10 MHz or higher, and a sensitivity ranging from 61% to 90% was found [14,17,18].

In the present study, differences between MRI images and US were observed in the values of sensitivity, specificity, and diagnostic accuracy in the closed and open mouth positions (joint movements assessment). 

In the study from Emshoff et al. [15], sensitivity was found to decrease from closed to open mouth position. This was attributed to the medial displacement of the disc after opening the mouth as the mandibular condyle and the glenoid cavity do not allow adequate ultrasound propagation, thus compromising the visualization of the articular disc.

Specificity increased from closed to open mouth position. The diagnostic accuracy was greater in the open mouth than in the closed mouth position, but in both positions the results obtained were acceptable. Similar results were found in studies by Landes et al. [14] and Jank et al. [16].

In the present study, the exam started positioning the transducer parallel to the Frankfurt plane while performing two scans: The first one in the rest position and the second scan with the mouth in maximum opening. Then, the position of the transducer was modified at an angle of 60°−70° with respect to the Frankfurt plane, and the two scans were repeated. 

In general, previous literature report that the position of the transducer can vary from horizontal (parallel to the zygomatic arch) to vertical (parallel to the mandible branch), thus providing different images of the TMJ in a transverse or coronal/sagittal plane. 

Actually, the image planes are rarely true transverse, coronal, or sagittal because they are almost always tilted and because the transducer is tilted during the examination in order to obtain a better visualization of the different components of the TMJ, especially the disc. This consideration does not apply to three-dimensional ultrasound, where the TMJ can be evaluated in different planes within the scan volume. Three-dimensional ultrasounds also had acceptable sensitivity and accuracy. 

In general, the present data show that interpretation of the images can be dependent on the operator. Therefore, experienced doctors are required to obtain reliable results and to connect US images to the recorded data from clinical and anamnestic exam. 

In the present study, a hand-carried US instrument was adopted that can be also employed in a dental office. From a general clinical point of view, the use of US offers some general advantages to dentists: the possibility of a rapid screening and allow carrying out a dynamic and direct investigation on the structure under examination or on the movement of muscles, tendons, joints, and ligaments, which is often essential for better understanding the physio pathological structure of the joint. It also allows a contextual contralateral comparative study. The survey is repeatable in a short time allowing monitoring of the pathology or follow-up of the therapies performed on the patient. The low cost of the examination compared to other investigation methods (CT and MRI) represents an additional advantage. By considering this, there is an increasing need to develop a standard operating protocol and the use of transducers of at least 10–12 MHz.

Furthermore, US is easy to use directly in the clinic without waiting to go to the diagnostic center, and it takes short period of time to obtain a response, which is not at the same level of the MRI, but it is always acceptable.

More advanced imaging such as MRI can be used after a positive screening with ultrasound to confirm TMD diagnosis if deemed necessary [25].

The following-up of TMJ alterations in dynamics is a significant advantage for making an accurate diagnosis, selecting an effective treatment method, and adequate assessment of recovery processes. All this may greatly enhance the quality of the dental practitioner’s work and treatment outcome of functional pathologies of the masticatory system [26].

The possible limitations of this exam are as follows: it should be performed by a healthcare professional; the operator should be aware of the anatomy of the TMJ; and, finally, to apply it in the diagnosis, the observation of images should also include clinical and anamnestic evaluations in order to perform an accurate diagnosis as TMJ disorders can be related to musculoskeletal disorders in other anatomical districts [27]. The limitation of this study is that the analyzed sample included few patients; a subsequent study showing the application of US in a larger sample would be appropriate.

## 5. Conclusions

The use of hand-carried US instrumentation in the diagnosis of TMD is useful and has clinical advantages over traditional MRI. The greater accessibility of the hand-carried US than MR is one of them. Thus, further research could test and promote the use of hand-carried US for the diagnosis and treatment of TMDs. It would be advantageous in terms of costs, accessibility, and easier monitoring of patients with TMJ disorders. 

As for all instrumental tests. if not corroborated by clinical and anamnestic data, the efficacy of US in identifying the position of the disc during the opening and closing movements appears limited compared to MRI.

Although US showed a lower diagnostic efficiency than MRI, it could still be useful for diagnostic purposes given some clinical advantages compared to MRI.

## Figures and Tables

**Table 1:** Difference between magnetic resonance and ultrasound.

	MRI		US	
	Sensitivity	Specificity	Sensitivity	Specificity
Identification of joint structures	97%	98%	77%	84%
Condylar position	98%	98%	82%	89%
Disc position	89%	90%	65%	82%
Joint dynamics	76%	76%	54%	56%

## Data Availability

Archived datasets analyzed and generated during the study are available via email after requesting them from the authors (via email).

## Supplementary Materials

The following are available online at https://www.mdpi.com/article/10.3390/mps4040081/s1, Table S1: Demographic and diagnostic data of the subjects included in the sample.

## Abbreviations

TMD	Temporomandibular Disorder
ID	Internal Derangement
TMJ	Temporomandibular Joint
MRI	Magnetic Resonance Imaging
US	Ultrasonography
ADD	Anterior Disc Displacement
MHz	MegaHertz
DD	Disc Displacement
JE	Joint Effusion
CE	Condylar Erosion
Se	Sensitivity
Spe	Specificity
Ac	Accuracy
PPV	Positive Predictive Value
NPV	Negative Predicted Value
DC/TMD	Diagnostic Criteria for Temporomandibular Disorders
CT	Computed Tomography
