# Supplementary material for: Hand-Carried Ultrasonography Instrumentation in the Diagnosis of Temporomandibular Joint Dysfunction
Source: Methods Protoc. 2021 Nov 6;4(4):81. doi: 10.3390/mps4040081 (PMC8629014; doi:10.3390/mps4040081)
Supplement: Supplementary file 1 [file mps-04-00081-s001.zip › mps-1334046-supplementary.pdf]

methods and protocols

MDPI

Article

Hand-carried ultrasonography instrumentation in the diagnosis of temporomandibular joint dysfunction.

Marco Severino [1], Silvia Caruso[1], Sofia Rastelli [1], Roberto Gatto [1], Tommaso Cutilli [1], Laura Pittari[2], Alessandro Nota[2], and Simona Tecco [2,*]

Table S1. Demographic and diagnostic data of the subjects included in the sample.

Patient	Gender	Age	Side	Click	Roar	Helkimo index *
1	M	38	left	-	+	II
2	F	19	left	+	-	II
3	F	26	right	+	-	II
4	F	32	left	-	+	II
5	M	43	left	+	+	II
6	M	31	right	-	+	II
7	F	28	right	-	+	II
8	M	36	left	+	-	II
9	F	33	right	-	+	II
10	F	57	left	+	+	III
11	M	49	left	+	+	III
12	F	24	right	+	-	I
13	F	37	right	+	-	II
14	F	46	right	+	-	III
15	M	39	left	-	+	II
16	M	29	right	+	-	I
17	F	33	left	+	-	I
18	F	38	right	-	+	II
19	F	44	right	-	+	II
20	M	52	left	+	+	III
21	M	31	left	-	+	II
22	F	35	left	+	-	II
23	M	42	right	-	+	II
24	M	46	left	+	+	I
25	F	39	right	-	+	II
26	M	30	right	-	+	II

27	M	29	left	+	-	I
28	F	41	right	-	+	II

* Helkimo index includes three sub-indices (anamnesis, dysfunction, and occlusal index) to evaluate TMJ dysfunction. The anamnesis index differentiates the patient into 3 levels (absent, medium, and severe symptoms) based on the symptoms of dysfunction reported during an interview. The dysfunction index differentiates the patient into 4 levels (absent, medium, moderate, and severe dysfunction) based on a functional evaluation of the mandibular range of motion, impairment, and pain on palpation or during movement. Its scoring range is from 0 to 25. The occlusal index differentiates the patient into 3 levels (absent, moderate, and severe occlusal disorders) based on the analysis of individual occlusion.

The Helkimo index differentiated the patient into 3 levels, that are Helkimo I = mild signs or symptoms, Helkimo II = moderate symptoms, and Helkimo III = severe symptoms.
